# Case report: Is exchange transfusion a possible treatment for metabolic decompensation in neonates with methylmalonic aciduria in the setting of limited resources?

**DOI:** 10.3389/fped.2022.926793

**Published:** 2022-07-25

**Authors:** Xiaoyu Cui, Na Li, Hong Xue, Fang Zhang, Jianbo Shu, Yang Liu

**Affiliations:** ^1^Department of Neonatology, Tianjin Children's Hospital/Tianjin University Children's Hospital, Tianjin, China; ^2^Graduate College, Tianjin Medical University, Tianjin, China; ^3^Tianjin Pediatric Research Institute, Tianjin Children's Hospital/Tianjin University Children's Hospital, Tianjin, China; ^4^Tianjin Key Laboratory of Birth Defects for Prevention and Treatment, Tianjin Children's Hospital/Tianjin University Children's Hospital, Tianjin, China

**Keywords:** exchange transfusion, hyperammonemia, metabolic decompensation in methylmalonic aciduria, primary hospital, neonate

## Abstract

Hyperammonemia is a serious complication of methylmalonic acidemia, with high mortality and permanent neurological sequelae in survivors. Primary hospitals are often the first admission hospitals for these children but are limited by their experience and facilities to provide rapid and effective treatment, increasing the risk of death in children with methylmalonic acidemia's metabolic crisis. In this report, we reported a case of a 7-day-old male neonate with decompensated methylmalonic acidemia, who underwent automatic peripheral arteriovenous exchange transfusion. The serum ammonia level of the boy decreased significantly post exchange transfusion. Therefore, we put forward the suggestion of exchange transfusion for hyperammonemia, in combination with medical therapy, in children with inborn errors of metabolism as an initial treatment option in primary hospitals if a rapid transfer to a center with dialysis facilities is not possible.

## Introduction

Methylmalonic acidemia (MMA) is a rare autosomal recessive disorder caused by different mutations either in the methylmalonic acid mutase (MMA) enzyme or in its coenzyme cobalamin. It is characterized by the abnormal accumulation of branched-chain amino acid metabolites, such as methylmalonic acid, malonic acid, 3-hydroxy propionic acid, and methyl citrate (1). Hyperammonemia is a neonatal emergency, with high mortality, and permanent neurological sequelae for survivors (2, 3). As a result, timely and effective treatment of this disease is crucial to reducing mortality and morbidity.

In this report, we reported a case of a 7-day-old male neonate with decompensated methylmalonic acidemia who underwent automatic peripheral arteriovenous exchange transfusion (ET). The serum ammonia level of the boy decreased significantly post ET. In addition, ET was introduced in the late 1940s to decrease mortality and morbidity due to kernicterus in hyperbilirubinemia and has become a universally performed technique in China (4). Moreover, ET has evolved into automated technology, making it safer and easier to operate.

## Case description

A male term infant, weighing 3.07 kg at birth, was admitted to the neonatal intensive care unit (NICU) of Tianjin Children's Hospital at 7 days of age because of poor feeding, lethargy, and hypoglycemia. This boy was born to a 37-year-old woman with gestational diabetes mellitus after a full-term pregnancy. Spontaneous rupture of membranes occurred 24 h before the vaginal, vertex delivery. Apgar scores were not provided.

On examination at admission, the weight was 2.71 kg, the rectal temperature was 36 °C, the blood pressure was 68/35 mmHg, the pulse was 128 beats per min, the respiratory rate was 36 breaths per min, and the oxygen saturation was 98%, while he was breathing ambient air. He showed an inactive response. Laboratory investigations showed severe metabolic acidosis (pH 7.373, pCO2 13.4 mmHg, pO2 130 mmHg, HCO3^−^ 7.8 mmol/l, and Beb −13.5 mmol/l), hyperammonemia (>1,000 ug/dl), elevated anion gap, electrolyte disturbance, and hypoglycemia (blood glucose level of 1.9 mmol/L). In addition, blood cell count showed decreased hemoglobin, white blood cells, and platelets. The remainder of the examination was normal.

Alarmingly, while waiting for the laboratory diagnosis, after the aggressive application of L-arginine, L-carnitine, and disaccharide lactulose to decrease the level of serum ammonia, combined with correcting electrolyte and fluid imbalances, there was no significant improvement in laboratory indicators, but the boy fell into a coma after 13 h of his hospitalization. At this time, urine and blood organic acid analyses suggested methylmalonic acidemia, which suggested that the neonate was in the decompensated phase of the disease. As a result, we performed a double-volume automatic peripheral ET for 2 h with a flow of 200 ml/h. After 26 min of ET, serum ammonia was dramatically decreased to 532 ug/dl, and other laboratory indicators improved significantly. Then, the patient was continued to be applied with L-carnitine to compensate for secondary carnitine deficiency, daily intramuscular vitamin B 12 injection (1.0 mg), and a specially designed formula (without Isoleucine, methionine, valine, and threonine) was given to ensure adequate energy intake on day 3. The boy continued to improve, and serum ammonia was returned to normal on day 7.

Genetic testing showed two heterozygous variants of the *MMUT* gene—located on chromosome 6p12—at c.626dupC (Lys210^*^) and c.682C>T (Arg228^*^), which further certified the diagnosis of methylmalonic acidemia. When assessed before discharge, the indicators of the child were significantly improved, and the physical examination of the neurological system was normal. In addition, there were no abnormalities found in auxiliary examinations, such as nuclear magnetic resonance of the brain, electroencephalogram, visual evoked potential, and brainstem auditory evoked potential, and the quality assessment of general movements was normal; then, the boy was discharged. At the first follow-up after 7 days of his discharge, the serum ammonia showed 92 ug/dl.

## Discussion

Hyperammonemia is a severe complication of MMA, with high mortality and permanent neurological sequelae in survivors, which may be caused by the inhibition effects of the accumulation of organic acids in the urea and tricarboxylic acid cycles (2, 3). In patients with MMA, the abnormal accumulation of propionyl-CoA inhibits the urea cycle by reducing the synthesis of N-acetylglutamate, an essential activator of carbamylphosphate synthetase (5). As a result, the ammonia produced by protein degradation cannot be detoxified in the liver by binding it to the urea cycle, resulting in high ammonia concentration in the blood (6). Moreover, the synthesis of succinyl-CoA in MMA is impaired, which facilitates the degradation of glutamine to produce α-ketoglutarate to improve the activity of the Krebs cycle activity, which contributed to chronic hyperammonemia (2). There are also some scholars who believe that because the low level of carnitine is related to the suppressed expression of urea cycle enzymes, secondary carnitine deficiency in MMA may lead to hyperammonemia (2, 7, 8).

In a study of 26 children with congenital urea synthesis errors, who survived neonatal hyperammonemia coma, Dr. Msall et al. (9) found that prolonged neonatal hyperammonemia coma was associated with brain damage and impaired intellectual function. Moreover, Dr. Corey et al. (10) noticed that the mortality increased when the blood ammonia concentration was >300 ug/dl, and the mortality was 85% when the peak blood ammonia concentration was >300 ug/dl. Therefore, appropriate and prompt therapy is paramount to improving outcomes.

For children with hyperammonemia, protein intake should be stopped immediately, and intravenous infusion of adequate calories, fluids, and electrolytes are the initial treatment. When it comes to medical therapy, L-carnitine is considered safe. It does not only compensate for secondary carnitine deficiency due to the loss of carnitine combined with organic acids in urine but also has antioxidant and anti-inflammatory effects, which may improve or prevent neurological damages caused by excessive ammonia (1, 11, 12). Besides, disaccharide lactulose, sodium benzoate, N-acetylglutamate analog, and so on are also used in the management of hyperammonemia (1, 2). Extracorporeal detoxification should be considered and prepared to start in neonates with a serum ammonia level above 400–500 μmol/l, or the neonates who do not respond to medical treatment within 4 to 6 h (1, 13). At present, continuous veno-venous hemodiafiltration (CVVHDF) is the prior choice recommended for extracorporeal detoxification in neonates and infants (1). Hemodialysis (HD) is another choice but is rarely used in newborns and infants due to technical challenges and related complications, such as hemodynamic instability, rapid fluctuation in intravascular volume status, and rebound of the blood level of toxic metabolites after dialysis (14), although Eisenstein et al. (14) demonstrated the safety and efficiency in newborns with inborn errors of metabolism. Additionally, peritoneal dialysis (PD) can be used as an alternative dialysis modality in low-resource settings (2). However, CVVHDF, HD, and PD are not available in most primary hospitals.

With the public awareness of the orphan disease and the popularization of neonatal MMA screening added to a huge population in China, MMA is an increasingly recognized disease. In addition, some researchers have shown that the incidence of MMA in China is significantly higher than that in other countries, which poses an additional challenge to China‘s health system (15). Unfortunately, many children with MMA decompensation are often first admitted at primary hospitals and would not be given timely and effective treatment due to the large distance to specialized centers. In addition, a study found that pre-dialysis coma duration exceeding 33 h was the limit invariably associated with a poor outcome (16). Therefore, a longer transfer time will likely result in a higher risk of death and a poor prognosis. In this report, we described a neonate with MMA metabolic decompensation successfully treated with ET and L-carnitine, which resulted in gradually decreased serum ammonia levels and clinically improved status of the child. Thus, we postulate that ET, in combination with medical therapy, could be a treatment option for MMA metabolic crisis in primary hospitals if rapid transfer to a specialized center with dialysis facilities is not possible.

In summary, we propose that ET, in combination with medical therapy, can be used by clinicians in primary hospitals as an initial and life-saving treatment option in decompensated MMA if a rapid transfer to a center with expert metabolic clinicians and dialysis facilities is not possible.

## Data availability statement

The original contributions presented in the study are included in the article/supplementary material, further inquiries can be directed to the corresponding authors.

## Ethics statement

The studies involving human participants were reviewed and approved by Ethics and Human Committee of Tianjin Children's Hospital. Written informed consent to participate in this study was provided by the participants' legal guardian/next of kin.

## Author contributions

XC, NL, and HX collected data and wrote and revised the first draft of the manuscript. XC, NL, HX, and FZ analyzed the data. JS and YL conceived the idea and reviewed the manuscript. All authors read and approved the final draft.

## Funding

This study was supported by the grant funded by Tianjin Key Medical Discipline (Specialty) Construction Project and the Project of Tianjin Municipal Health and Health Committee (No. ZC20120).

## Conflict of interest

The authors declare that the research was conducted in the absence of any commercial or financial relationships that could be construed as a potential conflict of interest.

## Publisher's note

All claims expressed in this article are solely those of the authors and do not necessarily represent those of their affiliated organizations, or those of the publisher, the editors and the reviewers. Any product that may be evaluated in this article, or claim that may be made by its manufacturer, is not guaranteed or endorsed by the publisher.
